# The Antitoxin Treatment of Diphtheria*Read before the Clinical Society of Maryland, January 18, 1895.

**Published:** 1895-03

**Authors:** L. F. Barker

**Affiliations:** Baltimore, Md.


### THE ANTITOXIN TREATMENT OF DIPHTHERIA.*

By L. F. BARKER, M.D.,

BALTIMORE, MD.

The antitoxin treatment is a final step in a long series of investigations. The principles underlying it are not new; the methods of immunization now in use are not unlike those used by Pasteur in his earlier work, to which he was undoubtedly stimulated by the success of Jenner's vaccination. After it was found out that the symptoms and lesions in the infectious diseases were dependent in the main on the toxic products of bacteria, it was soon discovered that the chemical toxins formed by the bacteria were capable, when introduced in gradually increasing doses into animals, of giving rise to an artificial immunity almost as certainly as the inoculation of the virus itself.

* Read before the Clinical Society of Maryland, January 18, 1895.

Those who were attacking the problems of immunity—that is to say, were endeavoring to discover what changes took place in the body of an individual during and after an infection, such as smallpox, which rendered him, after thorough recovery, practically insusceptible to a second attack—studied the fluids and tissues of the body before, during, and after an infection. These investigations led to the formation of two schools: first, that which believes the normal resistance offered against infection, and the immunity acquired by one attack or by artificial means depend upon certain properties of the blood-serum; and second, that which holds that the activity of the cells of the body accounts for the phenomena of both natural and acquired immunity. Dr. Nuttall showed that the blood-serum of an animal that had been immunized against anthrax, when injected into another animal, would kill more anthrax bacilli than the blood-serum of a susceptible animal. Other investigators proved that the use of the blood-serum from an immunized animal would, when introduced into another animal, protect it from infection with the same micro-organisms. Then Behring and his assistants demonstrated that the injection of the blood-serum of animals rendered artificially immune against diphtheria and tetanus would heal these infections, even after they were well started, in other animals. It was easier to understand how it would be possible to set up an artificial immunity against smallpox or typhoid fever than against diseases like diphtheria or pneumonia, for the latter are diseases from which an individual may suffer more than once. Immunity has to be looked upon as a relative term, and we may speak of a temporary and of a permanent immunity. An animal into which the bacterial toxins are injected has to suffer a reaction before it becomes immuned; a certain space of time must elapse before the antitoxins are formed. In the most successful method of producing artificial immunity, one begins by injecting small quantities of the well-diluted poisons. One of the most difficult problems in applying the serum therapy to human beings lay in obtaining a serum which contained the antitoxic substance in sufficient concentration, so that not too large quantities would have to be used for injection. Larger animals than those usually experimented upon had to be employed, and the horse has been for various reasons selected as most suitable. A small dose of diluted diphtheria toxins is at first injected into the region of the shoulder. The animal is somewhat disturbed and does not take its food as usual. After several days a second dose is administered, increasing doses producing less effect, until, after a period of from four to six months, the horse is rendered immune and the antitoxic strength of its serum may have attained a high degree. The serum is tested from time to time as to its antitoxic power, and when sufficient concentration has been reached the blood is drawn, the serum separated, standardized, and inclosed in flasks. Behring's so-called normal serum is of such a strength that one-tenth of one cubic centimeter of it will counteract, when injected with it into an animal, ten times the minimum amount of diphtheria poison which is fatal for a guinea-pig weighing three hundred grammes. One cubic centimeter of this normal serum is called an antitoxin unit. Serum No. 1 of Behring is sixty times as strong as this normal serum, serum No. 2 one hundred times as strong, and serum No. 3 one hundred and forty times as strong. In treating the disease, the earlier the antitoxin is given the better will be the result. Of the cases treated during the first two days, practically one hundred per cent. get well. At first, too small doses were given; now, not less than six hundred units (one flask of No. 1) are given as a beginning dose, and if the case be very severe, or be seen late, as much as sixteen hundred units may be given immediately. Within twenty-four hours after the injection, the pulse, as a rule, is slower, the temperature lowered, and the patient feels better in every way. If the cases are not seen until the third or fifth day, when the organs may already be seriously affected, it cannot be expected that the antitoxin will have such a beneficial effect; it can only counteract the poisons then present; it cannot repair the damage already done. A few relapses have occurred after its use, and some deaths, but these were not, it is claimed, in cases treated from the beginning. Very gratifying statistics come from Germany and France; the mortality rate has been markedly lowered. The disease, Behring states, is now absolutely within the control of the physician. It was thought at first that one-tenth of the ordinary healing dose would suffice to protect those who had been exposed to the disease from contracting it. But it is now recommended that one hundred and fifty units be injected as a prophylactic or immunizing dose. Some curious after-effects have followed its use, such as urticaria and erythematous eruptions, pains in the joints, sometimes accompanied by swelling, but in no instance were these symptoms of serious import. Laryngeal complications, it is stated, do not develop if the antitoxin has been used before they appear. It is claimed that tracheotomy is rarely necessary, and that intubation will answer in those cases where the larynx is indicated. The antitoxin is not to be looked upon as a direct chemical antidote, for it does not act against poison in the same manner that an acid neutralizes an alkali. For example, there is evidence in support of the view that the antitoxin acts indirectly by rendering the cells of the body capable of resisting the action of the toxins. The antitoxin for one disease may act to some extent in increasing the resistance of the body cells against the toxins of different origin. For instance, while the blood-serum of an animal rendered immune against snake poison has no antitoxic effect against the toxin of tetanus, yet an animal which is immunized against tetanus yields a serum which combats the toxic effect of snake poison, and there are other facts adduced which shake our confidence in the specificity of antitoxins. There may be to a certain extent an overlapping of the immunities. Diphtheria offers, as Buchner has pointed out, a better opportunity for the study of the effects of a new remedy than does tuberculosis; for, while the former approaches more nearly to a typical infection, the latter is almost a typical intoxication. Again, while tuberculosis runs a protracted course, as a rule, and is subject to spontaneous exacerbations and ameliorations, diphtheria is an acute process, terminating soon either in recovery or in death, and thus is a disease in which conclusions concerning the efficacy or futility of a given method of treatment may be speedily arrived at. Koch's tuberculin treatment differed from the antitoxin treatment of diphtheria in that in the former a glycerine extract of cultures of the tubercle bacillus was directly injected into the patient, there to set up a reaction, which, after a time, was to lead to the formation of healing substances; while in the latter the toxins of the diphtheria bacilli are injected into an animal, the animal suffers the reaction and builds the healing substances, and these are transferred ready-made to the human being. Should the new treatment of diphtheria prove to be as satisfactory as it promises, the outlook for the cure of infectious diseases in general is bright. We shall, however, be compelled to wait patiently until the bacteriologists, to whom all the credit of this new treatment is due, have perfected the arrangements for the application of the serum therapy to the other infectious diseases.

